# Child Sexual Abuse, Self-esteem, and Delinquent Behaviors During Adolescence: The Moderating Role of Gender

**DOI:** 10.1177/08862605211001466

**Published:** 2021-03-14

**Authors:** Amélie Gauthier-Duchesne, Martine Hébert, Martin Blais

**Affiliations:** 1 Université du Québec à Montréal, Québec, Canada

**Keywords:** child sexual abuse, gender differences, delinquency, self-esteem, adolescence

## Abstract

To reflect the complex phenomena of child sexual abuse (CSA), studies should examine possible gender specificities and explore potentially explanatory mechanisms. The current study aimed to test the moderating effect of gender in the mediated relationship between CSA, self-esteem, and delinquency during adolescence. A moderated mediation model was tested among a representative sample of 8,194 high school students (57.8% girls and 42.2% boys) age 14 to 18 in the province of Quebec in Canada. Results showed that self-esteem has an indirect effect on the relationship between CSA and delinquency. Gender (being a boy) was associated with a higher level of self-esteem and an increased risk of delinquent behaviors. Among victims of CSA, boys reported lower levels of self-esteem than girls, which was associated with an increased risk of displaying delinquent behaviors. Self-esteem may be an important target of intervention for sexually abused youth, especially for boys. Focusing on promoting positive self-esteem may also reduce the risk for male adolescents struggling with the deleterious consequences of delinquency.

## Introduction

Based on a worldwide meta-analysis, between 8% and 31% of girls and 3% to 17% of boys have been sexually abused prior the age of 18 (Barth et al., 2013). Child sexual abuse (CSA) is associated with multiple difficulties throughout life for both girls and boys. The magnitude and complexity of this public health issue demonstrate the necessity for studies to be conducted to better understand the experiences of girl and boy victims, in the hopes of adapting prevention efforts as well as medical, legal, and psychological interventions.

The adolescent period of life will be examined in this study. This developmental period is one of transition, where different specific issues can be experienced (e.g., family distancing, first romantic relationships, new social experiences), potentially affecting the trajectories of victimization. Adolescence also corresponds to the crystallization and expression of socially gendered norms, making it possible to observe issues related to masculinity. In addition, compared to pre-adolescent children, there is a higher level of delinquent behaviors during adolescence (Achenbach & Rescorla, 2001); this is the outcome of interest in the current study. The adolescent age group (14–18 years old) includes young people that are often concentrated in delimited places (e.g., schools, youth centers), making the deployment of prevention and intervention measures more optimal.

Over the past 30 years, there has been a proliferation of studies conducted on CSA victims to identify the deleterious consequences of interpersonal trauma related to sexual abuse. Reviews of the literature and prospective studies clearly show that CSA is associated with post-traumatic stress disorder (PTSD), lower self-esteem (Fergusson et al., 2013), depressive and anxiety symptoms (Gardner et al., 2019), and even physical health problems (Daigneault et al., 2017). Also, victims of CSA are at higher risk of developing externalizing symptoms, such as conduct disorder (Maniglio, 2015) and violent and delinquent behaviors (Kozak et al., 2018). All these consequences of CSA can affect both men and women throughout their lives (Maniglio, 2009).

There is a growing interest among the scientific community surrounding the specific outcomes in boy victims. However, too few studies have focused on male sexual victimization and the possible gender specificities related to the outcomes associated with CSA (Depraetere et al., 2018). For example, in his systematic review on the association between CSA and conduct disorder, Maniglio (2015) reported that only 8 of the 36 studies investigated included controls for gender. In the general population, higher levels of delinquent behaviors are observed, being more persistent in boys compared to girls (Zheng & Cleveland, 2013). The question becomes: Is this disparity also present among CSA victims, or would it even be amplified? Some studies suggest that boy victims display more externalizing difficulties than girl victims (Gauthier-Duchesne et al., 2017; Kozak et al., 2018), while others observe no such gendered difference (Maniglio, 2015; Soylu et al., 2016).

Although the prevalence of CSA is lower among boys, they appear to nevertheless experience significant long-term consequences. A recent study with a sample of men found that CSA contributed more to difficulties in adulthood when compared to the other forms of childhood abuse and maltreatment (Turner et al., 2017). In their large sample of men (*n* = 14,564), the authors compared four groups: no maltreatment, child maltreatment without CSA, CSA only, and CSA with other types of child maltreatment. The last two groups demonstrated higher odds of developing mental disorders and higher rates of suicide attempts compared to men with a history of child maltreatment without CSA. The disparity in the literature regarding gendered specific outcomes, as well as the severity of the consequences experienced by male victims of CSA, help to underline the importance of studying this population.

### Male Victims of CSA at Higher Risk of Delinquent Behaviors

Recent data confirm the relationship between CSA and delinquent behaviors. According to several studies, this association remains present even after accounting and controlling for other forms of maltreatment experienced (Hébert et al., 2019; Kozak et al., 2018). Therefore, CSA seems to make a unique contribution to the emergence of delinquent behaviors.

It is less clear, however, whether this type of behavior is more prevalent among boys or girls with a history of CSA. At first glance, theoretical foundations on masculinity may help guide answers to this question. Raewyn Connell (2005) conceptualized masculinity in four dynamics: hegemony, subordination, complicity, and marginalization. The first dynamic, which represents the cultural domination of masculinity (e.g., power, money, independence), describes domination over subordinate men. The domination of heterosexual men and the subordination of homosexual men is the most frequently cited example. But this relationship of domination/subordination can also be observed between male sexual offenders and their male victims. Men with a history of sexual victimization may feel vulnerable and passive (Price-Roberston, 2012). Aggressive and delinquent behaviors may thus appear to some men (Connell, 2005) as a way to restore their self-image and masculinity (Depraetere et al., 2018) that have been otherwise threatened or damaged by the abuse (Alaggia & Millington, 2008).

In a systematic review, Maniglio (2015) reported that for victims of CSA there is between a 2- and 12-times increased risk of having conduct disorder. Most of the studies (7 out of 8) that have compared victims by gender found similar rates of conduct disorder in male and female youth victims. For their part, Soylu et al. (2016) examined the medical and judicial reports of 1,250 sexually abused youth (248 boys) aged 18 or under in Turkey. Using chi-square analyses, this study found no significant differences in the prevalence of conduct disorder between girls (4.0%) and boys (5.9%). Despite the absence of significant difference, it remains important to note that these studies focused on the clinical prevalence of the DSM-5 diagnosis of conduct disorder (American Psychiatric Association, 2013), which refers to a “continuing pattern of behavioral disregard for the rights of others and social norms for acceptable conduct” (Berkout et al., 2011, p. 503).

When a broader definition of delinquent behaviors is used, different findings, more consistent with the theoretical assumptions previously made, are observed. Delinquent behaviors refer to antisocial behaviors, divided into two subtypes: nonviolent (e.g., damaging or destroying something that belongs to someone else, stealing something) and violent (e.g., the use or threat of use of a weapon, hurting someone physically; Zheng & Cleveland, 2013). In their study on the effects of CSA on delinquent and violent behaviors in adolescence, Kozak et al. (2018) used a list of different behaviors, which they then dichotomized (absence or presence of violent and delinquent behavior). They found that compared to boys, adolescent female victims of CSA were less likely to engage in violent and delinquent behaviors. In a study of 447 CSA victims (128 boys) age 6 to 12, Gauthier-Duchesne et al. (2017) also observed that boys display higher scores of externalizing problems (continuous scores of aggressive and rule-breaking behaviors) when compared to girls. Therefore, male victims of CSA appear to be more likely to display delinquent behaviors, but further studies are needed to better understand this phenomenon. For example, it would be relevant to compare different types of delinquent behaviors between boy and girl victims of CSA. Only observing the proportion of boys and girls who meet the diagnostic criteria of conduct disorder impedes insight gained from nuancing the specificities of gender differences. For example, girls may be more susceptible to using tactics of bullying and cruelness, while boys may more often initiate physical fights (Berkout et al., 2011).

Studies should also explore the potential mechanisms by which CSA is associated with delinquent behaviors in boys and girls. In 1985, decades before the proliferation of studies on CSA, Finkelhor and Browne published a conceptual model where four traumagenic dynamics helped to describe the diversity of consequences associated with CSA. One of these dynamics, stigmatization, refers to the comments and behaviors of the abuser (or other people from the family and community) that hurt and blame the child. This dynamic is particularly associated with shame and guilt. According to Finkelhor and Browne (1985), the consequences of this stigmatization can lead to delinquent behaviors, such as drug and alcohol abuse and involvement in criminal activities. Stigmatization could also be related to a lower self-esteem. After perceiving negative and degrading attitudes toward victims (from the abuser, the family, or the community), people who have been sexually abused may conclude that they have less value than others (Finkelhor & Browne, 1985). The concept of self-esteem can thus be a mediator of interest in explaining the association between CSA and delinquency, considering that low self-esteem has been empirically established as a consequence associated with CSA (Fergusson et al., 2013; Maniglio, 2009).

As for the relationship between self-esteem and delinquent behaviors, the link between the two has been studied for decades. As early as the 1970s, researchers concluded that self-esteem has an impact on delinquency rates, but the reverse connection was not found (Rosenberg & Rosenberg, 1978). Youth with lower self-esteem may engage in delinquent behaviors first to reject groups or norms that devalue them, and second to benefit the approval and respect from delinquent subgroups (Kaplan, 1975). This could be observable among sexually victimized boys who search for a restoration of their self-esteem through behaviors valued by culturally dominant norms of masculinity, including aggressive and delinquent behaviors (Connell, 2005).

In a diverse sample of young adolescents, Donnellan et al. (2005) found a strong relationship between low self-esteem and delinquency, which was not explained by confounding variables (age, gender, supportive parenting, and academic achievement). Despite some methodological issues in the literature (e.g., variations in the conceptualization of self-esteem, differentiation between crime and delinquency), a recent meta-analysis on 42 studies confirms that self-esteem is negatively associated with delinquency (Mier & Ladny, 2018).

The relationship between CSA, self-esteem, and delinquent behaviors is thus relevant to explore given that this mediator can be a target of clinical intervention for young victims. This relationship may also be different for girls and boys (Hébert et al., 2019). Including mediating variables, such as self-esteem, can therefore provide a better understanding of gender differences among CSA victims, not limited to the observation of potential differences.

### The Present Study

Despite a growing interest in male sexual victimization, male victims remain insufficiently studied (Depraetere et al., 2018). More efforts should therefore be made to better understand their experiences, and possibly to offer more appropriate support. Studies should also take into account a greater diversity of delinquent behaviors and integrate mediating variables, including explanatory mechanisms in the relationship between CSA and this type of behavioral problems. Even if it is widely known that boys of the general population engage in more aggressive and antisocial behaviors than girls, it remains to be documented whether this also applies among CSA victims. Documenting this issue, as well as exploring self-esteem as a potential mediator, could provide concrete avenues of interventions for practitioners working with young CSA victims, who may also be juvenile offenders.

The aim of the present study was to explore gender differences in the relationship between CSA and delinquent behaviors among an adolescent population. The specific objectives were to (a) compare the proportion of boy and girl victims of CSA on different delinquent behaviors and (b) test a model of mediation between CSA, self-esteem and delinquent behaviors, integrating gender as a moderating variable. We hypothesized that boys would display more delinquent behaviors than girls, and this effect would be greater among CSA victims.

## Method

### Participants and Procedure

The sample was drawn from the *Youths’ Romantic Relationships Project*. Overall, 8,194 adolescent students (57.8% girls and 42.2% boys) in the province of Quebec in Canada completed a questionnaire. The participants were aged 14 to 18 years old and attended a private or public high school. The majority of the sample spoke French at home (75.4%), lived with both their parents (63.1%), and had parents who identify themselves as Canadians (78.0%). The parents’ ethnicity of the other participants is North African or Middle Eastern (4.4%), Asian (3.9%), European (3.6%), Caribbean (3.6%), Latino-American (3.5%), African-American (1.9%), from First Nations or Inuit (.5%) or other ethnic groups (.5%).

The survey was completed from 2011 to 2012 through a one-stage stratified-cluster sampling of high schools. To obtain a representative sample of students in grades 10 through 12, schools were randomly selected from an eligible pool from the Quebec Ministry of Education for the 2010 to 2011 school year. These schools were classified into strata according to metropolitan geographical area, status of school (public or private), teaching language (French or English), and social-economic deprivation index. A weight for each respondent was used, which was represented by the inverse of the probability of selecting the given grade in the respondent’s stratum in the sample multiplied by the probability of selecting the same grade in the same stratum in the population.

Among the 131 schools that were randomly solicited, a total of 34 school administrations (28 public and 6 private) agreed to participate. A research assistant went in the classrooms and students were free to accept or refuse to complete the questionnaire, which was fulfilled during the class period. The acceptance rate was 99%. A list of resources (e.g., telephone helplines, websites, regional health services) and the contact information of school personnel (e.g., psychologist, sexologist, social worker, mental health practitioner, nurse) were provided to the students. The project was approved by the Human Research Review Committee of the Université du Québec à Montréal.

### Measures

#### Socio-demographic characteristics.

Participants were asked if they were a girl (0) or a boy (1). They were also asked about their month and year of birth, the spoken language at home, and if they and/or their parents were born in Canada.

#### Child sexual abuse.

CSA includes sexual acts committed against a person under the age of 18, without his or her real consent, by an adult or another person under the age of 18 in a position of power or authority (Mathews & Collin-Vézina, 2019). Although not unanimous, this definition is widespread in the literature and circumscribes the exact nature of maltreatment by excluding other forms of sexual victimization (e.g., sexual harassment, sexual dating violence). To measure the occurrence of CSA, two items were used in this study. The first mentioned unwanted touching (“Have you ever been touched sexually when you did not want to, or have you ever been manipulated, blackmailed, or physically forced to touch sexually?”). The second one referred to unwanted sexual activities involving penetration (“Has anyone ever used manipulation, blackmail, or physical force, to force or obligate you to have sex [including all sexual activities involving oral, vaginal, or anal penetration]?”). These items were developed by Finkelhor et al. (1990) in an American national study and used in a national survey on the prevalence of CSA among adults living in the province of Quebec (Hébert et al., 2009). A dichotomized score was created so that each participant was considered as either sexually abused (1) or not (0).

#### Self-esteem.

A 4-item version (Statistics Canada, 2007) of the *Self-Description Questionnaire* (Marsh & O’Neill, 1984) was used to measure adolescents’ self-esteem. This scale’s version derives from the National Longitudinal Survey of Children and Youth (NLSCY; Statistics Canada, 2007). The data obtained in this national survey support the psychometric properties of the scale, with an adequate internal consistency (*α* = .82) and correlations among items ranging from .38 to .64 (Ferro & Boyle, 2013). For each item (e.g., “Overall, I have a lot to be proud of.”), the response scale ranged from 0 (false) to 4 (true) and the total score ranges from 0 to 16. The internal consistency of the scale in the present study was high (*α* = .86).

#### Delinquent behaviors.

To assess delinquent behaviors, a 6-item questionnaire, also from the NLSCY, was used (Statistics Canada, 2007). For each item, the adolescents were asked how many times they exhibited the described behavior in the past 12 months (e.g., “In the past 12 months, approximately how many times have you intentionally damaged or destroyed something that didn’t belong to you?”). The total frequency varies from 0 to 36. A dichotomized score was also used in the descriptive analysis to compare the participants who had a behavior at least once versus those who never had such behavior. The internal consistency was *α* = .69.

### Statistical Analysis

A representative sample was collected among Quebec high school adolescents in grades 10 to 12. The cluster-stratified sampling had 8 strata, 34 clusters (one for each school). Complex sample commands were used in the analyses described below. A weighted sample of 6,531 youths resulted and is used for the descriptive analyses.

For our first objective, a *t*-test was conducted to compare the mean scores of delinquent behaviors between male and female adolescent victims. Then, for each of the six delinquent behaviors, categorical descriptive analyses were performed to explore if there was a higher proportion of boys or girls who endorse each item as occurring at least once. Considering the complex-sampled design of the survey, the adjusted *F-*statistic was used, which is a variant of the second-order Rao-Scott chi-square statistic (Rao & Scott, 1981). The SPSS 26 software was used for these descriptive analyses.

**Figure 1.**
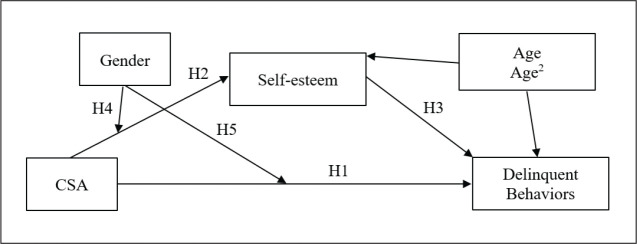
Conceptual moderated mediation model of CSA, self-esteem, and delinquent behaviors, with gender as moderator.

Second, a moderated mediation model illustrated in Figure 1 was conducted to achieve the second objective. The variables included were as follows: CSA as the independent variable, self-esteem as the mediated variable, delinquent behaviors as the dependent variable, and gender (being a boy) as the moderated variable. Gender was considered moderating both the CSA-self-esteem path and the CSA-delinquent behaviors path.

The model postulated that: CSA would directly increase delinquent behaviors (H1); CSA would indirectly increase delinquent behaviors through the decrease of self-esteem (H2); the decrease of self-esteem would increase delinquent behaviors (H3); being a boy would exacerbate the effects of CSA on self-esteem (H4) and on delinquent behaviors (H5). A path analysis approach was used to simultaneously test these hypotheses in a moderated mediation model (Hayes, 2015).

In addition, age of the participants and quadratic effect were included as covariables to control for the possible increase of delinquent behaviors in older adolescents. No fit indices were relevant because the model was saturated. For a few participants, values were missing for the dependent variables or for all variables except the dependent one, so the model includes 7,721 participants. To perform this model, analyses were conducted using M*plus* 8.0 (Muthén & Muthén, 1998–2017) with these commands: MLR (maximum likelihood estimation with robust standard errors), Full Information Maximum Likelihood to handle with the missing values, and 10,000 iterations.

## Results

### Descriptive Statistics on Gender Differences Among CSA Victims

Overall in the sample, 14.9% of adolescent girls and 3.9% of adolescent boys reported having been sexually abused. A *t*-test conducted among this subsample of CSA victims indicated a significant difference between boys and girls on the delinquent behaviors scale (*t*_(26)_ = −4.35; *p* <.001). Boys reported having more delinquent behaviors (*M* = 7.02; *SE* = .73) than girls (*M* = 3.75; *SE* = .21). A higher proportion of boys indicated engaging in delinquent behavior at least once in the last year for 4 of the 6 items: staying out all night without permission (*F*_(1, 26)_ = 6.71; *p* = .016), intentionally damaging or destroying something (*F*_(1, 26)_ = 28.64; *p* <.001), getting into a fight (*F*_(1, 26)_ = 83.39; *p* <.001) and carrying a weapon (*F*_(1, 26)_ = 17.50; *p* <.001). The percentages for each item are presented in Table 1. No gender differences were found for 2 items (“Have you ran away from the place you where live?” and “Have you stolen something?”).

**Table 1.** Proportions in Percentage of Delinquent Behaviors for Girl and Boy Victims of CSA.

Delinquent Behaviors	Girls(*n* = 532)	Boys(*n* = 96)	*F*_(1, 26)_	*p*
1. Have you stayed out all night without permission?	36.3%	52.2%	6.71	.016
2. Have you run away from the place you live?	17.6%	20.6%	.43	.520
3. Have you intentionally damaged or destroyed something that didn’t belong to you?	32.2%	56.9%	28.64	<.001
4. Have you stolen something?	39.1%	46.5%	2.67	.115
5. Have you gotten into a fight with someone with the intention of seriously injuring them?	18.7%	48.2%	83.39	<.001
6. Have you carried a weapon for the purpose of defending yourself or using it in a fight?	10.6%	26.0%	17.50	<.001

*Note*. The percentages represent the proportions of girl and boy victims of CSA who endorse each delinquent behavior as occurring at least once in the last year.

### Moderated Mediation Model

**Figure 2.**
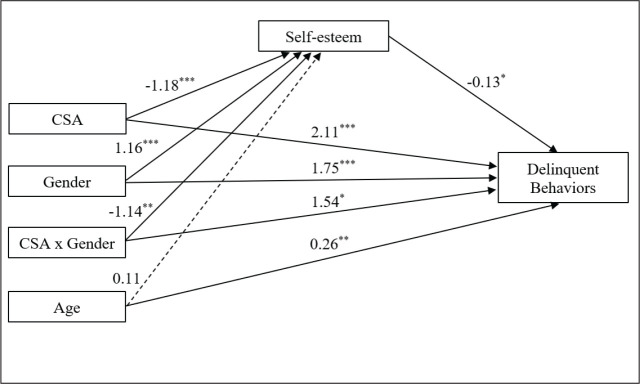
Statistical moderated mediation model of CSA, self-esteem, and delinquent behaviors, with gender as moderator.

*Note*. Regression coefficients from the estimated model are written.

Table 2 shows, for the total sample, the matrix correlation of the variables in the moderated mediation model. The statistically tested model is shown in Figure 2, and the coefficients in Table 3. CSA was directly associated with delinquent behaviors (H1; *β* = 2.11, *p* <.001), and indirectly with self-esteem (H2; *β* = −1.18, *p* <.001 and H3; *β* = −.13, *p* <.001). The moderating role of gender was also statistically significant in the model. Being a boy exacerbated the effects of CSA on self-esteem (H4; *β* = −1.14, *p* = .003) and on delinquent behaviors (H5; *β* = 1.54, *p* = .036). All of the observed effects were in the expected directions. The age of the participants was positively associated with delinquent behaviors (*β* = .26, *p* = .009), but not with self-esteem (*β* = .11, *p* = .099). In the correlation matrix, the quadratic effect of age was significantly correlated to delinquent behaviors. However, when integrated in the model, this covariable was no longer associated with any of the endogenous variables. So, the quadratic effect of age was removed from the tested model. The model explains 4.4% of the variance in self-esteem and 8.8% of the variance in delinquent behaviors.

**Table 2.** Summary of Correlations, Means, and Standard Errors for the Variables in the Moderated Mediation Model.

Variables	1	2	3	4	5	*M*	*SE*
1. CSA						–	–
2. Gender	–.18***					–	–
3. Self-esteem	–.14***	.17***				11.52	.12
4. Delinquent behaviors	.16***	.17***	–.10***			2.35	.14
5. Age	.07***	.01	.02	.08***		15.86	.11
6. Age squared	.07***	.01	.02	.08***	.99***	252.44	3.47

*Note*. *M* = Mean. *SE* = Standard error.

***p < .001.

**Table 3.** Unstandardized Coefficients of the Moderated Mediation Model Estimating Self-esteem and Delinquent Behaviors.

	Self-esteem				Delinquent Behaviors		
	Coeff.	*SE*	*p*		Coeff.	*SE*	*p*
CSA	−1.18	.15	<.001		2.11	.23	<.001
Self-esteem					−.13	.02	<.001
Gender	1.16	.11	<.001		1.75	.17	<.001
CSA × Gender	−1.14	.38	.003		1.54	.74	.036
Age	.11	.06	.099		.26	.10	.009

*Note*. Coeff. = Unstandardized regression coefficients. SE = Standard Error.

Full lines illustrate significant paths, and the dotted line shows nonsignificant path.

Gender has been coded as 0 = Girl and 1 = Boy.

*p < .05. **p < .01. ***p < .001.

## Discussion

This study explored gender differences in the relationship between CSA and delinquent behaviors among a representative sample of adolescents age 14 to 18 years old. First, the proportion of adolescent male and female CSA victims who engaged in different delinquent behaviors was compared. The results showed that, for four items out of six (staying out all night without permission; intentionally damaging or destroying something; getting into a fight; carrying a weapon), a higher proportion of boy victims engaged in the delinquent behavior at least once in the last year. No gender differences were observed for the two other delinquent behaviors measured (running away from the place of residence; stealing something). Second, a moderated mediation model linking CSA, self-esteem, and delinquent behaviors was tested, with gender as moderator. All the statistical hypotheses were confirmed; CSA was associated with delinquent behaviors through lower self-esteem scores. Being a boy was associated with more delinquent behaviors and this effect was greater among CSA victims.

The literature on juvenile delinquency clearly establishes the tendency that boys, compared to girls, show higher rates of delinquent behaviors (Zheng & Cleveland, 2013), but it was uncertain if this difference was also observable among CSA victims. The present study shows not only that this difference is present, but also that it is accentuated among adolescents with a history of CSA. Adolescent male victims of CSA were more likely to engage in delinquent behaviors than their female counterparts. In other words, being a boy and having been sexually abused were associated with an increased risk of delinquent behaviors. This effect was more important when these two conditions were combined. Our results are consistent with those obtained in other studies on adolescents (Kozak et al., 2018) and school-age children (Gauthier-Duchesne et al., 2017) who were sexually abused.

However, this conclusion cannot be extrapolated to other constructs related to juvenile delinquency, such as rates of crimes and conduct disorder. Maniglio (2015) and Soylu et al. (2016) found no difference between boy and girl victims of CSA in the proportion of conduct disorder. The measure of juvenile delinquency used (e.g., a scale of delinquent behaviors vs a diagnosis of conduct disorder) may influence the observation of gender differences. Among CSA victims, boys and girls may be equally likely to meet the diagnostic criteria of conduct disorder. On the other hand, our results revealed that boy victims report more delinquent behaviors than girls. It is possible that boys are more likely to engage in some delinquent behaviors without meeting the DSM diagnosis. A scale of delinquent behaviors may better detect variability through the observation of multiple antisocial behaviors and their frequencies among young victims.

Delinquent behaviors can be divided in two subtypes: nonviolent and violent. In examining the behaviors for which there were differences between girls and boys, we noticed a higher proportion of victimized boys engaging in violent delinquent behaviors (getting into a fight and carrying a weapon). While boys in the general population reported more violent delinquency than girls (Zheng & Cleveland, 2013), the results of the present study suggest that this could also be true for CSA victims. Boy victims may tend to engage more in violent behaviors, which embody hegemonic masculinity (Connell, 2005). For example, nearly one out of two male adolescent victims (48.2%) reported getting involved in a fight with the intention of seriously injuring the other person. Trying to demonstrate physical strength and domination in a fight could be a way for male adolescent victims to assert a particular type of masculinity that may have been eroded during CSA, a masculinity that is emblematic of the domination and cultural ideals of hegemony (Connell, 2005).

Gender also had a moderating effect on the association between CSA and self-esteem. In the total sample population, boys had higher levels of self-esteem. However, among the CSA victims, we observe the opposite tendency: boy victims seem to have lower self-esteem than girl victims. These two moderating effects of gender may be explained by the ways in which some male adolescent victims cope with CSA. Thus, one hypothesis would be that the dynamic of stigmatization, which may lead to delinquent behaviors and low self-esteem (Finkelhor & Browne, 1985), is more salient for boys. Stigmatization may also be exacerbated by the fear for boys of being perceived as homosexual, since past studies clearly show that boys are more likely to be abused by a same-sex perpetrator (Gewirtz-Meydan & Finkelhor, 2020). The lack of adequate support after disclosure (if the abuse were disclosed) could also amplify the stigmatization. Indeed, the majority of male victims of CSA state that they remember the disclosure as a negative experience, like not being believed or having received threats (Gagnier & Collin-Vézina, 2016). Boys may be more coerced to act out sexual gestures during the episodes of CSA and half of male adolescent victims are assaulted by a minor (Gauthier-Duchesne et al., 2017). This may reinforce the false belief that boys engage in sexualized behaviors but are not CSA victims. In short, these issues in the literature on sexual victimization of boys and men may contribute to the emergence of delinquent behaviors among victimized boys, both in demonstrating hegemonic masculinity (Connell, 2005) and in benefiting from the valorization of their masculinity by their peers (Kaplan, 1975).

In addition to looking for the approval of a group, delinquent behaviors can respond to an adaptive function. For example, getting involved into a fight can enhance one’s sense of masculinity. Staying out all night without permission can allow for the temporary escape from a violent family environment. While these behaviors may meet a certain need for adaptation, some violent delinquent behaviors can seriously harm other individuals. Different spheres of influence, including family and community, should offer skills to young victims of CSA that are more adaptive in the long run and which are likely to be less harmful, both personally and environmentally. For example, strengthening self-esteem could be a target of intervention, considering that this variable is associated with delinquent behaviors among the participants of the study.

### Implications for Practice

The results of this study clearly demonstrate an association between CSA, self-esteem, and delinquent behaviors during adolescence, and the role of gender. We might think that girls are more likely to show internalized symptoms following CSA, but that below their “tough guy” façade, self-esteem of victimized boys is also damaged. In terms of clinical implications, practitioners should help youth strengthen the assets that make them proud, according to their interests. First, practitioners should therefore assess delinquent behaviors (forms and frequency). For the victims who reveal a concerning level of delinquent behaviors, the treatment should be adapted to this issue. For example, the Trauma-Focused Cognitive Behavioral Therapy (TF-CBT; Cohen et al., 2017) was found efficient in fostering recovery in CSA victims. Among the different applications of the treatment, the one including 16 therapy sessions without a trauma narrative component demonstrated a greater reduction of externalizing behaviors in children (Deblinger et al., 2011). These improvements in externalizing behaviors may be related to more time being devoted to the parent training component (Deblinger et al., 2011). It would be interesting to evaluate such an approach for adolescent CSA victims who engage in delinquent behaviors.

Relative to girls, boys are less likely to disclose CSA (Hébert et al., 2009; Okur et al., 2017). Furthermore, boy victims of sexual violence are less likely to seek services (Alaggia & Millington, 2008; Fernet et al., 2019). It is therefore possible that boys with serious delinquent problems end up in the justice system and youth centers, but practitioners (psychologists, social workers, judges, police officers) may be unaware of their CSA history. Professionals should be better informed of the possible causes that can lead to delinquent behaviors (Kozak et al., 2018). CSA and other adverse childhood experiences should also be a part of the evaluation process for young offenders.

To help these young people, the Attachment, Self-regulation, and Competency framework (Blaustein & Kinniburgh, 2018) can be implanted in juvenile justice centers (Collin-Vézina et al., 2019). Strengthening self-esteem and secure attachment for trauma victims, in addition to providing more appropriate emotional regulation skills and self-identity building, may help to reduce delinquent behaviors (Collin-Vézina et al., 2019). Undoubtedly, the juvenile justice system would benefit from taking a trauma-informed approach. Implementing gender-responsive interventions in the justice system could also help to reduce the risk of recidivism among victimized boys and girls, but more research is needed (Kerig, 2018).

### Limitations and Future Studies

Some limitations of the present study should be mentioned. First, the cross-sectional design used cannot establish a causal relationship between CSA, self-esteem, and delinquent behaviors, nor can it verify if the gender differences are maintained in adulthood. Second, the scale use to assess delinquent behaviors comprised of only six items. Third, the variance explained by the tested model remains modest. To overcome these limitations, future studies should propose a longitudinal design, use a more refined measure of delinquent behaviors, and include other variables that may interact in the relationship between CSA and delinquency. For example, other traumas could be included to confirm the unique contribution of CSA to delinquent behaviors.

The lack of recognition of gender diversity is an important limitation to discuss. The study questioned gender in a binary fashion, leaving no opportunity for persons from gender minorities to be represented. It is imperative that future studies allow respondents to select their own gender identity (Fraser, 2018). In addition, the present study highlighted differences between boys and girls who were victims of CSA but did not describe the intra-gender nuances. The use of a person-centered approach would be relevant to better understand the various experiences of boys. Nevertheless, the study has several strengths. The representative sample makes it possible to generalize the results to all the youth aged 14 to 18 attending high school in Quebec. It also highlights some issues that boy victims of CSA may face, a population that is underrepresented in the literature.

## Conclusion

Delinquency is an outcome associated with CSA to which more attention should be paid, especially among boy victims. While engaging in delinquent behaviors can serve gendered purposes such as restoring self-esteem and masculinity, it also has deleterious consequences not only for the youth themselves but also for people around them. More appropriate coping alternatives should therefore be offered to them, including tools to fortify their self-esteem.

## Authors’ Note

This article was submitted as partial recognition of the doctoral degree in sexology of the first author.

## Author Biographies

**Amélie Gauthier-Duchesne**, MA, is a PhD candidate at the Department of Sexology at the Université du Québec à Montréal. Since her master’s degree, her research interests focus on the short-term outcomes associated with child sexual abuse in school-age and adolescent boys. She also has an interest in exploring the experiences of survivor disclosure.

**Martine Hébert**, PhD, is a full professor in the Department of Sexology at the Université du Québec à Montréal and the Tier 1 Canada Research Chair in Interpersonal Traumas and Resilience. Her research focuses on child sexual abuse and dating violence among adolescents and young adults.

**Martin Blais**, PhD, is a full professor in the Department of Sexology at the Université du Québec à Montréal. He is the Co-chairholder of the Research Chair on Homophobia. His research interests are sexual diversity, sexual health, intimate relationships, and the sociology of sexuality and intimacy.
